# Honesty the Best Policy

**Published:** 1861-08

**Authors:** 


### HONESTY THE BEST POLICY.

THE late Duke of Buccleugh, in one of his walks, purchased a cow in the neighborhood of Dalkeith, which was to be sent to his palace on the following morning. The Duke in his morning dress espied a boy early ineffectually attempting to drive the animal forward to its destination. The boy not knowing the Duke, bawled out to him, "Hie, mun, come here, an' gie's a han' wi' this beast." The Duke walked on slowly, the boy still craving his assistance, and at last, in a tone of distress, exclaimed, "Come here, mun, an' help us, an' as sure as onything I'll gie you half I get!" The Duke went and lent a helping hand. "And now," said the Duke, as they trudged along, "how much do ye think ye'll get for this job?" "I dinna ken," said the boy, "but I'm sure o' something, for the folk up by at the big house are gude to a' bodies." As they approached the house, the Duke disappeared from the boy, and entered by a different way. Calling a servant, he put a sovereign into his hand, saying, "Give that to the boy who brought the cow." The Duke having returned to the avenue, was soon rejoined by the boy. "Well, how much did you get?" said the Duke. "A shilling," said the boy, "an' there's half o' it to ye." "But you surely got more than a shilling," said the Duke. "No,"

said the boy, "as sure as death that's a' I got—an' d'ye no think it's plenty?" "I do not," said the Duke, "there must be some mistake; and as I am acquainted with the Duke, if you return, I think I'll get you more." They went back, the Duke rang the bell, and ordered all the servants to be assembled. "Now," said the Duke to the boy, "point me out the person that gave you the shilling." "It was that chap there with the apron"—pointing to the butler. The butler confessed, fell on his knees, and attempted an apology; but the Duke indignantly ordered him to give the boy the sovereign, and quit his service instantly. "You have lost," said the Duke, "your money, your situation, and your character by your covetousness; learn henceforth that honesty is the best policy." The boy by this time recognized his assistant in the person of the Duke; and the Duke was so delighted with the sterling worth and honesty of the boy, that he ordered him to be sent to school, kept there, and provided for at his own expense.—Anon.

